# “Team chemistry” through chemistry lenses: interdisciplinary science or a metaphorical conundrum?

**DOI:** 10.3389/fpsyg.2015.00038

**Published:** 2015-01-28

**Authors:** Edson Filho

**Affiliations:** Behavioral Imaging and Neural Dynamics Center, University of ChietiPescara, Italy

**Keywords:** sport psychology, group dynamics, team chemistry, team sports, team performance

A Google search on “team chemistry” returns over 443,000 entries (October 2014) usually denoting some sort of team process, such as cohesion, shared mental models, and collective efficacy. Practitioners (e.g., athletic coaches and business managers) often emphasize the importance of team chemistry for optimal performance. For instance, former NFL quarterback and current business executive Roger Staubach noted that “In any team sport, the best teams have consistency and chemistry.” Researchers in performance psychology also allude to the notion of “team chemistry” when discussing exceptional teams (Levine, 1994; Gershgoren et al., 2013).

Although practitioners and researchers refer to team chemistry as an antecedent of team expertise, there has been neither philosophical nor conceptual debate on whether chemistry principles may indeed inform performance and sport psychology research and practice. Therefore, the question that motivates me to write this commentary paper is: “*What if scholars would indeed seek inspiration in microscopic chemistry principles in order to better understand macroscopic team dynamics*?” Of note, mixing “soft” and “hard” sciences is common in the pursuit of interdisciplinary research. Applied philosophers and psychologists have long tried to establish connections between group processes and the natural world (i.e., “Associationist School”). Recently, psychologists have employed concepts from physics, including quantum theory, to study decision-making in social contexts (Busemeyer et al., 2006). Specific to chemistry, Green (2012) uses history to discuss fundamental organic chemistry principles, while Nobel Laureate Harold Kroto (2005) wrote about how art and design inspired his research on chemistry.

Although I do not have a “set-in-stone” response to the question I propose herein, I open debate and share insights on whether, and how, general chemistry concepts may be useful to performance and sport psychologists interested in team dynamics. Specifically, I comment on two overarching chemistry concepts that emerged in several Nobel Laureates' presentations during the 63rd Lindau Nobel Laureate Meeting (LNLM) in chemistry. During this week-long scientific gathering 34 Laureates discussed how chemistry (and its disciplines, such as organic, inorganic, and biochemistry) has been used to inform research and practice in various domains of human interest including social matters such as education and politics. First, I discuss whether the concept of “inter-action” from a chemical viewpoint may promote parsimonious research and meta-cognitively inform applied work in psychology. Second, I comment on “structure means function” by illustrating the notion of catalysis in chemical reactions, particularly discussing how leaders may serve as “catalysts” of team processes and performance. A remark from a Nobel Laureate in chemistry follows my discussion, offering an independent analysis of the idea discussed herein. Finally, a senior sport psychology researcher and a human movement scientist share their reactions to the idea of “team chemistry through chemistry lenses.”

## Inter-action

For centuries, chemists have combined various substances to create new compounds leading to a newer or more optimized process. Foremost, a chemists' assumption is that the *inter-action* coupling between two or more independent agents will lead to better, more specific, or more complex chemical structure. Similar to chemists, psychologists are interested in studying interactions among behaviors, bio-psycho-social processes, and neural mechanisms (Bandura, 1997; Hanin, 2007). From a meta-cognitive standpoint, psychologists could discuss team member interactions borrowing ideas from various chemical reactions described in the chemistry literature. For instance, the principles of *single displacement* (A + BC → AC + B) and *double displacement* (AB + CD → AD + BC) could be used to study intra-team coordination in team sports. In effect, sport analysts have long debated why “star players” often perform better (e.g., greater number of assists and goals scored) when playing for their club team versus their national team. Part of the answer to this question may rest on understanding how high-performing (*strong links*) unique dyadic and triadic intra-team associations (e.g., Xavi↔Iniesta↔Messi in Barcelona football club) are created from the complementary skills of two or more players. Therefore, at times, rather than recruiting one star player, coaches should recruit (or focus on developing) a high-performing network of two or more players.

The notion of single and double displacement could also be used to explore the “too-much-talent-effect” (Swaab et al., 2014), which occurs when a team comprised of stars fails to play to its potential because the individual team members do not interact in a successful manner (*weak links*). Altogether, borrowing ideas from chemical interactions can be useful in studying the cyclical development of *team coordination* and *shared mental models* in team sports, similar to how psychologists have used the physics notion of small-world networks to inform research on brain networks. In this regard, there is consensus that different inter-team interactions lead to the development of myriad shared declarative, procedural and strategic forms of knowledge (Mohammed et al., 2010). At the LNLM, Jean-Marie Lehn emphasized the importance of multivalent interactions in the establishment of “dynamic adaptive coordination networks” at the supramolecular level. To Lehn, it is the ability of “supramolecular species to exchange their components” that allows for the development of highly sophisticated chemical systems in general (e.g., transport systems) and *catalysis recognition* in particular. Thus, could sport psychologists examine whether leaders serve as “catalysts” to improve group dynamics and performance in sport teams?

## “Structure means function”: catalysis recognition and team performance

Throughout the LNLM, the Laureates discussed how “structure means function,” particularly in regards to catalytic processes in biochemistry. Various natural catalytic processes occur through conformational changes involving two or more reactants (e.g., an enzyme and respective substrate), with distinct yet complementary structural characteristics (Green, 2012). Catalysis works by lowering the activation energy required for a given reaction to proceed. The “catalyst” provides an alternative chemical pathway for the reaction, usually by increasing the selectivity or reaction rate, or by decreasing the entropy in the system. There are also negative catalysts (i.e., *inhibitors*) that function to decrease the rate of a chemical reaction.

Similar to chemistry, “structure means function” can be applied to team sports. For instance, in soccer different systems and formations of play (4-3-3; 3-5-2; 5-4-1) are aimed at defensive or offensive tactical functions. The notion of an “isomer” (a molecule with the same chemical formula but that differs from another by the rotation of a single bond) from conformational analysis in chemistry could help performance analysts to explore how altering the position of a single player can significantly affect team performance.

Scholars in the “soft” sciences have also borrowed the idea of catalysis when discussing the role of transformational and peer leadership in team performance (Stanley et al., 2008; Venus et al., 2013; Filho et al., 2014). Transformational leaders promote change among their followers and within their organizations. To this extent, based on the overarching notion of catalysis, Venus et al. (2013) observed that leaders' emotional states (i.e., enthusiasm) prime followers' regulatory focus, which in turn generates high follower performance in regards to a shared team goal. In terms of peer leadership, sport psychologist Hanin (2007) noted that “in team sports, the major focus and concern of the coach are the emotional states of the goal-keeper and key-players (leader and sub-leaders) who affect the emotional dynamics of the entire team” (p. 37). Filho et al. (2014) also observed that peer leaders serve as “catalysts of team performance” by modeling confidence and communicating information during high-pressure situations. Overall, borrowing ideas from chemistry and other “hard” disciplines already exists in some capacity, even if at the basic conceptual level. Such exchanges of ideas and principles may continue to grow, as psychologists discuss hybrid concepts (e.g., psychogenetic, social neuroscience, DNA ancestry testing) that are made possible by mixing the “soft” and “hard” sciences.

## Final thoughts

I considered writing this commentary after interacting with several scholars during the 63rd LNLM. I invited one Nobel Laureate interested in cross-domain collaboration to share his reaction and insights on the idea of studying group dynamics “through chemistry lenses.” A senior sport psychologist and a human movement scientist who have integrated principles from different fields in their research also share their reactions on the idea discussed herein. Researchers and practitioners at large are invited to reflect on whether “team chemistry” will remain a metaphorical notion or whether it may evolve to a real exchange between chemists and applied psychologists.

## Reply from harold kroto—nobel laureate in chemistry

This is an interesting analysis of the significance of the word “Chemistry” which has developed a positive meaning in societal interaction apparently separate from the somewhat pejorative term “chemical” when applied to say food. In fact, the key creative process in chemistry is synthesis in which two or more known compounds are brought together in a reaction to produce a new and hopefully valuable compound. A favorite example of mine is the reaction of carbon dioxide and water in a vine to produce a carbohydrate which in the presence of an enzyme results in alcohol and finale wine. Thus the term chemistry as probed in this article is a perfect term to describe the sort of interactive intellectual environment needed to engender “Creativity” with a Capital C.

## Reply from gershon tenenbaum—sport psychologist researcher

The author raises the question whether principles from chemistry can account for human behaviors in a team context. This might be the case had people been comprised of molecules which produce artificial intelligence-like actions. However, one should consider that humans (e.g., “molecular entities”) in a team do not interact with each other as chemical entities do, but rather as entities with unique personalities, bio-psycho-social needs, and sets of cultural values. Furthermore, the author mentions that leadership within a team can be viewed within a framework of a chemical catalyst, which affects the team positively or negatively. One should keep in mind that energy produced in chemical reaction may differ substantially from energy produced by human entities. Members within a team may have opposite intentions, may belong to more than one sub-group within a team, or may select more than one leader (“catalyst”) for different tasks which ought to be accomplished. These are some thoughts to consider when transposing the principles of one domain to another.

## Reply from maurizio bertollo—human movement scientist

Teams are often paralleled to an organism in which different parts work together for a common goal. Analyzing team dynamics through “chemistry lenses” allows us to see the leader of a team like an “enzyme” that “catalyzes” reactions of an organism. The leader, like an enzyme, can work through anabolic (building team dynamics) and catabolic (destructing negative dynamics) pathways. For instance, if the leader is viewed as a “functional group” [e.g., carboxyl (-COOH)], s/he could eliminate (i.e., catabolism) other reactants that are not useful for the team. Overall, the idea expressed in this paper is an interesting way to study team dynamics in sport settings in respect to inter-actions and structure-function within a team. Our goal in sport sciences is to find the right pathways and catalysts to speed up reactions and inter-actions, while developing group anabolic and catabolic processes to improve team dynamics and performance.

### Conflict of interest statement

The author declares that the research was conducted in the absence of any commercial or financial relationships that could be construed as a potential conflict of interest.
